# Ecology of the Scorpion, *Microtityus jaumei* in Sierra de Canasta, Cuba

**DOI:** 10.1673/031.011.8601

**Published:** 2011-07-07

**Authors:** Franklyn Cala-Riquelme, Marco Colombo

**Affiliations:** ^1^ Centro Oriental de Ecosistemas y Biodiversidad (BIOECO) y Museo de Historia Natural “Tomás Romay” Enramadas #601, esq. Barnada Santiago de Cuba, 90100, Cuba; ^2^ Liceo Scientifico Statale A. Tosi, Busto Arsizio, Varese, Italy

**Keywords:** Cuban forests, environmental factors, relative abundance

## Abstract

An assessment of the population dynamics of *Microtityus jaumei* Armas (Scorpiones: Buthidae) on the slopes south of Sierra de Canasta, Guantánamo Province, Cuba show an increase in activity over the year (≤ 0.05). The activity peak is related to the reproductive period from June to November. The abundance of scorpions was significantly related to density of the canopy and thickness of the substrate.

## Resumen

Se hace un reconocimiento de la dinámica poblacional de *Microtityus jaumei* Armas (Scorpiones: Buthidae) en la vertiente sur de la Sierra de Canasta, Guantánamo, Cuba observándose a lo largo del año un incremento en la actividad (≤ 0.05). Este incremento guardó relación con el periodo de reproducción el cual sucedió entre los meses de junio y noviembre. La abundancia de los escorpiones estuvo relacionada con la cobertura vegetal y el grosor de la hojarasca.

## Introduction

Scorpions are a small group of about 2000 species of nocturnal terrestrial arthropods. During the day they seek shelter beneath rocks or logs, in cracks or burrows that they dig into the substrate, or beneath the loose outer layers of many plant shrubs and trees (Polis 1990). They are solitary and rather stationary in nature; thus they are often encountered in their shelter, which they only leave to feed and reproduce (Polis 1990; Lourenço 2000). Scorpions allow opportunities for ecological, conservation, and biogeographic study (Polis 1990; Prendini 2005).

The ecology of scorpions of the Caribbean and Americas is poorly understood. The classic monograph on the biology of scorpions by Polis (1990) does not discuss any aspect of the biology, ecology, or population density of the genus *Microtityus* (Scorpiones: Buthidae). A recent (2008) survey of literature on scorpions showed that all existing studies concern taxonomic work. Ecology is the least known aspect of scorpions of any kind in the world, perhaps because they are dangerous and often coexist with highly venomous snakes (Polis 1990).

The genus *Microtityus* Kjellesvig-Waering, consisting of the subgenera *Parvabsonus* Armas 1974 and *Microtityus*, is found in the Neotropical region in the Caribbean and Amazonian subregion (Fet and Lowe 2000; Morrone 2001). The genus *Microtityus* includes a group of small-sized scorpions; they share morphological similarities and preference for relatively rare and inaccessible habitats and thus have not been a frequent object of either ecological or biological study. The study presented here was conducted to assess the activity of a population of *M. jaumei* Armas over the course of one year (January 2005 to February 2006) and to assess the relationships between relative abundance and environmental variables in the study area.

## Materials and Methods

### Study area

**Figure 1.**
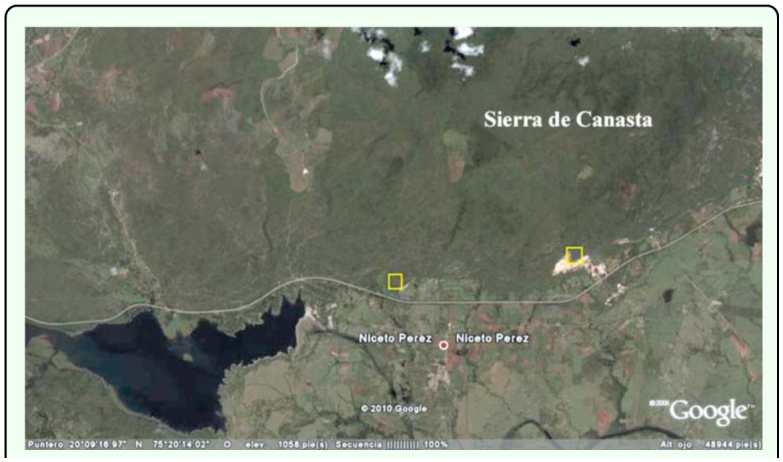
Study area. Sierra de Canasta, Guantánamo, Cuba. The approximate area of study is shown in the yellow squares. High quality figures are available online.

Specimens were collected over a year in three areas on the slope south of Sierra de Canasta (20° 09′ 43″ N - 75° 21′ 46′ W) that is within the jurisdiction of the municipality of Niceto Pérez, Guantánamo Province (Figure 1). It is a mountainous terrain of relatively recent geological origin, early to late Pleistocene according to Iturralde-Vinent (1969, 1978) that structurally consists of anticlines and monoclines that do not surpass 500 m elevation. The presence in some regions of marine erosion scarps and coastal marine deposits is a clear indication that the sea occupied or reached the area. Iturralde-Vinent (1969, 1978) and Kartashov et al. (1981) propose that this area was once a plain and periodically emerged during the periods corresponding to the lower PliocenePleistocene and part of late Pleistocene. Presently, the vegetation of the area has been reduced, to a large extent, to a semi-deciduous forest terrain and secondary pastureland; although the secondary scrub patches that can be found at some sites often extend considerably.

Specimens were collected in various habitats, which were classified as:

BSM 1 (semi-deciduous forest at 100–250 m elevation) characterized by a greater solar input and less vegetation cover (62% vegetation cover) and by a 2 – 8 cm thick layer of leaf litter.

BSM 2 (semi-deciduous forest at 250–100 m elevation) had a vegetation cover of 80% in some areas.

MXS (xenomorphic heath) was found on the marine erosion scarps and had a greater abundance of silts.

PAS (pastureland) constitutes areas with the lowest levels of vegetation cover (12%) due to the extensive grasses found in this habitat.

### Environmental data

Environmental data were collected as follows: thickness of the substrate, canopy density, and specimen position with respect to the ground, as well as time of day and sampling period. These data were collected monthly for one year, from February 2005 through January 2006, for each of the collection locations. Ten measurements were taken at random in each area by means of marked ruler and measuring rod. Using this approach the thickness of the substrate was classified as follows: uncovered to minimal cover (0–5 mm), covered (5–10 mm), and covered (greater than 10 mm). The canopy density calculation was made using a manual densimeter at the same ten locations in each area as previously measured and 15 samples were taken. On this basis, the canopy was evaluated according to arbitrary criteria and grouped as follows: highly isolated (< 25%), thin (25–50%) and thick (> 50%). The position of individual specimens in the substrate was then classified as, Type I (dorsal position with respect to the soil), Type II (upright/perpendicular to ground), or Type III (in soil).

### Collection of samples

Twelve monthly collections, of two days each, were undertaken between February 2005 and February 2006. Specimens were collected by hand so that that the species could be systematically monitored with the purpose of determining the current state of the populations and conservation status of the scorpiofauna in the area. Since all ecosystems were examined, a major effort was made to acquire representatives of the greatest number of species. Factors that suggest differences among ecosystems may affect distribution and composition of the scorpiofauna, so transects were established to address the possibility of shifts in the scorpiofauna (Berovides and Tadeo 1999). An *a priori* 4 × 4 m (16 m^2^) grid was used for the terrestrial plots, and the methods of Berovides and Tadeo (1999) were used for the study of populations and communities of arachnids.

### Statistical analysis

The statistical analysis was carried out using SSPS (version 12.0). The normality and homogeneity of variances were first tested and the data were ranked in both cases. To check if the number of specimens collected in each area differs in relation to environmental factors, the Kruskal-Wallis test was used with alpha = 0.05. To compare the number of specimens collected in the areas of higher elevation versus lower elevation the MannWhitney test was used with an alpha-value of 0.05. In order to verify whether there is a relationship between these factors and the number of specimens collected in each area, the Spearman Rank Correlation was used with n = 4.

## Results

*M. jaumei* individuals were observed in the study area foraging near their microhabitat (under rocks, trunks, and among in the forest litter decomposition). The greatest numbers of individuals were present in the semideciduous forest, followed by scrub and secondary xerophytic grassland; individuals, mostly juveniles, were frequently sighted in the transitional vegetation zones. Under normal moisture *M. jaumei* was found clinging to stone surfaces and other objects making them difficult to detect because of their cryptic coloration. Under unfavorable conditions (severe drought) they were most commonly found beneath the leaf litter or inside crevices in the ground. Once disturbed, specimens would assume a cataleptic posture (apparent death), which sometimes lasted from seconds to several minutes (mean = 4 min.). The presence of groups of individuals of the same species was often noted, and it was not uncommon to find two or more individual specimens (adult and juvenile) together. The maximum number found together was 11 (including two females with their respective litters of 5 and 7 juveniles, respectively), under a stone 18 × 11 cm in size; three individuals clung to the stone and the rest were on the ground. *M. jaumei* was also found living syntopically with *Rhopalurus junceus* (Herbst) (Buthidae). It was found with *Cazierius asper* Teruel 2006 (Scorpionidae) in BSM 1, BSM 2, and MXS; in cohabitation with *Centruroides gracilis* (Latreille) (Buthidae) in BSM 2; and sympatrically with *Centruroides anchorellus* Armas (Buthidae) in BSM 1 and BSM 2.

### Seasonality

**Figure 2.**
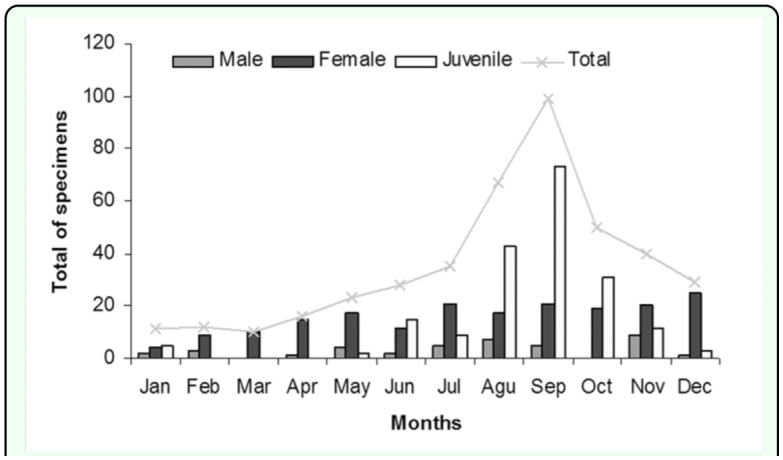
Seasonality of the scorpion population of the south slope of Sierra de Canasta, Cuba (January 2005 to February 2006), with the total number of individuals collected by month. The columns represent the number of males, females, and juveniles collected by month. High quality figures are available online.

The months of May (rain) and November (dry) turned out to yield the highest number of individuals per unit of sampling effort (between 23 and 99 individual specimens recorded during the period), representing 81% of the individual specimens seen and recovered. During much of the study period mature males were found, with the largest number during the rainy season (from late May until early November). In the subsequent months (dry season), the number of scorpions tended to decrease in number, with the period after October (November and December) being the most active as well as shortly before the start of the rainy period (April). The greatest numbers of females were in the months of July (0.11), September (0.11), and October (0.10). The lowest values were for the months of January (0.02) and February (0.04). Furthermore, a greater number of juveniles were detected in the months of August (immature) and September (immature) (Figure 2).

### Relative abundance in the four study areas

**Figure 3.**
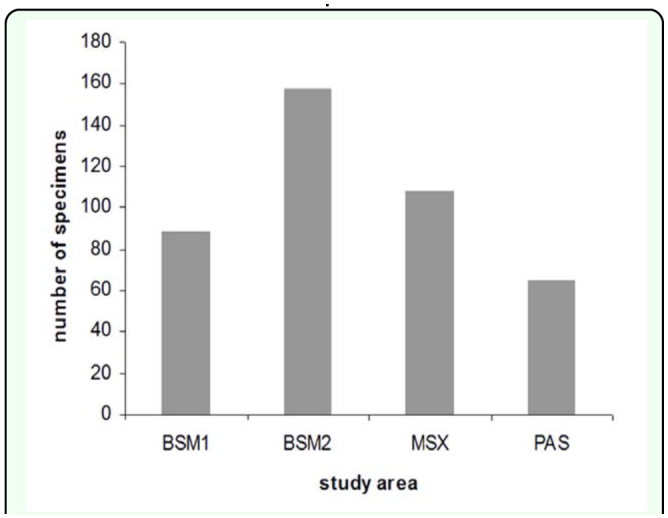
Relationship between number of specimens and study area. (Kruskal-Wallis: P< 0.05). Semideciduous forest at 100–250 m elevation (BSM 1), semideciduous forest at 250–400 m elevation (BSM 2), xenomorphic heath (MXS), and pastureland (PAS). High quality figures are available online.

**Figure 4.**
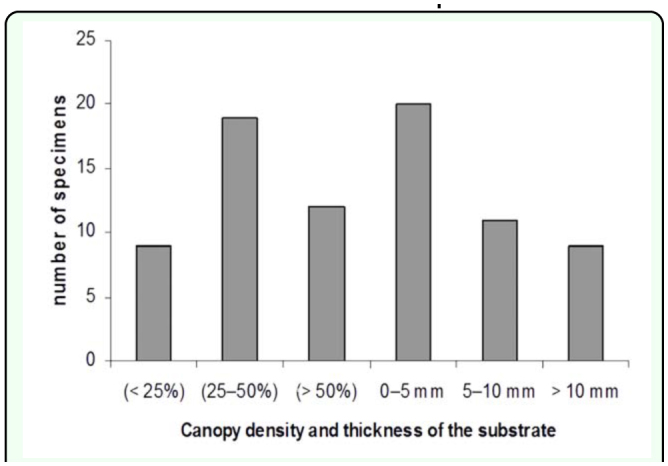
Relationship between canopy density and area of study (Kruskal-Wallis: P< 0.05, n = 40). Highly isolated (< 25%), thin (25–50%), and thick (> 50%). Uncovered to minimal cover (0–5 mm), covered (5–10 mm), and covered (greater than 10 mm). High quality figures are available online.

Individuals collected showed a tendency towards heterogeneous distribution in the four areas studied (Kruskal-Wallis, χ^2^ = 344,553; df = 3; P< 0.05) (Figure 3). The areas that had an effect of continuous succession (BSM 2 MXS-BSM 1 - PAS) showed a close similarity (Mann-Whitney, P < 0.05). Areas 1 and 3 were found to have significantly higher values (Mann-Whitney, U = 0,000, P < 0.05), which may be due to the type of habitat and the availability of existing microhabitats. The thickness of the substrate (litter layer) turned out to be different in the four areas (Area 1: 1.6 cm; Area 2: 2.14 cm; Area 3: 0.7 cm; Area 4: 0.4 cm; Kruskal-Wallis, χ^2^ = 18981; df = 1; P < 0.001 n = 40) (Figure 2). The canopy density result also differed in the four areas (Area 1 79.8%, Area 2 61.3%, Area 3 42.7% Area 4 27.1%; Kruskal-Wallis, χ ^2^ = 10785; df = 2; P< 0.05, n = 40) (Figure 4). The Spearman rank correlation found the influence of environmental factors on the increment of specimen number (coefficient of thickness of the substrate = 0,338, P < 0.05; coefficient of canopy density = -0,338, P< 0.05) that was proved to be highly significant when correlating the number of specimens to area of study (correlation coefficient of area = 0,873, P< 0.001) (Table 1). Soil type was the same in the four areas. The number of scorpions collected varied with the time of day and the season. With respect to time of day and position, it was observed that in the early hours of the day the scorpions were found in the Type I position; subsequently, as the day progressed this gradually changed to position Type II and finally Type III, during the warmer hours. During dry periods, a greater number of specimens were found in position Type III. Rainfall and temperature did not influence scorpion abundance in the study areas, except that populations increased during the rainy season.

## Discussion

The *M. jaumei* population of the southern slope of La Sierra de Canasta is rather stable throughout most of the year, but shows a remarkable increase during the rainy period (July–October). Activity peaks from the end of August into early November, during which time the greatest number of litters and pregnant females were found. Even so, males and females were found sharing the same micro-habitat throughout the entire year. This phenomenon occurs with greater frequency in the months leading up to the rainy period, during which time we commonly found groups of up to three pairs exploiting the same micro-habitat. This is correlated with the fact that fertilized females were found from August to November of 2006.

**Table 1.**
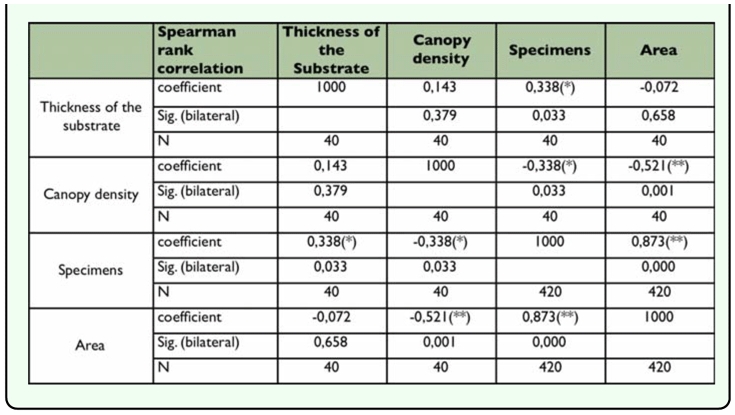
Spearman rank correlation between environmental factor (canopy density and thickness of the substrate), study area and specimens number.

The sit-and-wait foraging strategy of this species consists of remaining immobile, waiting for prey. This foraging strategy type does not require the scorpion to move around (Polis 1990), leading us to conclude that most of scorpions collected in this study were in a search of partners, not prey.

A similar studied was done by Corey and Taylor (1987). Pitfall traps were used to collect near Orlando, Florida, during one year, sampling every two months. Only one species was collected, *Centruroides hentzi* (Buthidae). This is an errant forager but also showed an increase in activity in July and September, that is, apparently, the reproductive period. Another study was done by Yamaguti and Pinto-da-Rocha (2003) on *Thestylus aurantiurus* (Bothriuridae) in the Parque Estadual da Serra da Cantareira, Brazil. In this case, September through November is the apparent reproductive period.

The reproductive period in scorpions varies according to the species. Some South American scorpions such as *Tityus bahiensis* (Buthidae) do not exhibit a well-defined reproductive period, instead remaining active throughout the year (Matthiesen 1968). There is information on several species of the Bothriuridae. The reproductive period of *Bothriurus bonariensis* is from November to February, that of *B. flavidus* is from November to January, and those of *Urophonius iheringii* and *U. brachycentrus* are from May to September. However, differences in the *Thestylus aurantiurus* reproductive period may be related to climatic differences at the different localities. The beginning of the reproductive period of the Argentinean species of Bothriurus coincides with the beginning of the local warm, wet season. In Cuba, previous studies of *Microtitys fundorai* and *M. trinitensis* (Buthidae) coincided with the beginning of the rainy period of July or August. For these populations, the beginning of the reproductive period is August to November although in general terms, the minimal climatic variability exhibited in the tropics (from the Tropic of Cancer to the Tropic of Capricorn) is known to favor reproductive activity throughout the entire year (see Polis 1990).

There is a great difference in activity between the sexes in the *M. jaumei* population of the southern slope of Sierra de Canasta. Many more females were captured than males (Figure 2), probably due to method used and male reproductive activity. Using manual active collection produces different results from pitfall traps. If pitfall traps are used, the expected number of captured scorpions would be higher and the expected number of males would be closer to the number of females (since the sex ratio of *M. jaumei* is ♂1:♀5). However, seasonal activity probably would not be as evident as in the collection with pitfall traps. It is evident that the population of *M. jaumei* in the southern slope of Sierra de Canasta possesses a reproductive season from August to November (Figure 2).

### Influence of environmental factors on abundance

Individuals of the *M. jaumei* population from the southern slope of Sierra de Canasta apparently prefer habitats at higher elevations. This may be because shelters in lower areas can be flooded in the rainy season. The two areas with a higher number of collected scorpions (1 and 3) are located at a higher elevation and farther from water sources. On the other hand, the two areas with fewer collected scorpions (2 and 4) are located in places at a lower elevation. These areas are close to water sources that are flooded in the rainy season. According to Polis (1990), some scorpion species seek specific environmental conditions. Namibian scorpions use several places as a shelter including simple holes in the soil and under tree bark (Lamoral 1979). Harington (1978) verified that *Cheloctonus jonesii* (Liochelidae) examines a large area before beginning to dig its burrow. Additionally, in *Urodacus* there are differences among species in the choice of place, format and structure of burrows (Koch 1978). Many factors influence the spatial distribution of scorpions. Important physical factors include temperature, precipitation, soil or rock characteristics, stone or litter cover, and environmental physiognomy.Temperature and precipitation are probably the most important determinants of general geographical range (Koch 1977, 1981; Newlands 1978; Prendini 2001, 2005). Some species reach their highest densities only in areas with extensive ground cover from rocks, logs, or other vegetation litter (Smith 1966; Koch 1978; Warburg and Ben-Horin 1978; Prendini 2001). Evidently, trees and the resulting litter often occur in areas characterized by a lower probability of flooding than in adjacent areas (Koch 1978). The canopy density and the thickness of the substrate were the most important environmental factors for *M. jaumei*, suggesting that this species of “bark scorpions” may be associated with dead vegetation (Stahnke 1966).

## Abbreviations:

BSM I,: semi-deciduous forest at 100–250 m elevation;
**BSM 2**,: semi-deciduous forest at 250–400 m elevation);
**MXS**,: xenomorphic heath;
**PAS**,: pastureland

## References

[bibr01] Armas LF (1974). Escorpiones del Archipielago Cubano. II. Hallazgo del genero *Microtityus* (Scorpionida: Buthidae) con las descripciones de un nuevo subgénero y très nueva especies.. *Poeyana*.

[bibr02] Armas LF (1982). Algunos aspectos zoogeográficos de la escorpiofauna antillana.. *Poeyana*.

[bibr03] Armas LF (1984). Escorpiones del archipiélago cubano. VII. Adiciones y enmiendas (Scorpiones: Buthidae, Diplocentridae).. *Poeyana*.

[bibr04] Armas LF (1988). *Sinopsis de los escorpiones antillanos*..

[bibr05] Armas LF (2002). Alacranes de Republica Dominicana. *Centruroides nitidus* (Thorell, 1876) y *Microtityus lantiguai* Armas and Marcano Fondeur 1992 (Scorpiones: Buthidae).. *Revista Ibérica Aracnología*.

[bibr06] Armas LF (2002). Redescubrimiento del alacran *Microtityus dominicanensis* SantiagoBlay (Scorpiones: Buthidae) de Republica Dominicana.. *Revista Ibérica Aracnología*.

[bibr07] Berovides V, Tadeo R (1999). *Manual de métodos de conteo en animales terrestres*..

[bibr08] Claro VA (1986). *Conferencias de biogeografia*..

[bibr09] Corey DT, Taylor WK (1987). Scorpion, pseudoscorpion and opilionid faunas in three central Florida plant communities.. *Florida Scientist*.

[bibr10] Fet V, Lowe G, Fet V, Sissom WD, Lowe G, Braunwalder ME (2000). Family Buthidae C.L. Koch, 1837.. *Catalog of the Scorpions of the world* (1758–1998).

[bibr11] Gonzalez-Sponga MA (1970). Record del genero *Microtityus* para Vénézuéla. II. *Microtityus biordi* (Scorpionida: Buthidae), nueva especie para el Sistema de la Costa en Vénézuéla.. *Monografías Científicas Augusto Pi Suñer*.

[bibr12] Gonzalez-Sponga MA (2001). Aracnidos de Venezuela. Seis nuevas especies del genero *Microtityus* (Scorpionida: Buthidae) del sistema Montanoso de la Costa.. *Boletín de la Academia de Ciencias Físicas, Matemática y Naturales*.

[bibr13] Harington A (1978). Burrowing biology of the scorpion *Cheloctonus jonesii* Pocock (Arachnida: Scorpionida: Scorpionidae).. *Journal of Arachnology*.

[bibr14] Iturralde-Vinent M (1969). Principal characteristics of the Cuban Neogene stratigraphy.. *American Association of Petroleum Geologists Bulletin*.

[bibr15] Iturralde-Vinent M (1978). Los movimientos tectónicos de la etapa de desarrollo platafórmico de Cuba.. Academia de Ciencia Cuba, Información Científico Técnica.

[bibr16] Koch LE (1977). The taxonomy, geographic distribution and evolutionary radiation of Australo-Papuan Scorpions.. *Records of the Western Australian Museum*.

[bibr17] Koch LE (1978). A comparative study of the structure, function and adaptation to differents habitats of burrows in the scorpion genus *Urodacus* (Scorpionida, Scorpionidae).. *Records of the Western Australian Museum*.

[bibr18] Koch LE, Keast A. (1981). The scorpions of Australia: aspects of their ecology and zoogeography.. *Monographiae biologicae*.

[bibr19] Lourenço WR, Cuéllar O (1994). Notes on the geography of parthenogenetic scorpions.. *Biogeographica*.

[bibr20] Lourenço WR, Huber D, Cloudsley-Thompson JL (1999). Notes on the postembryonic development of two species of Microtityus Kjellesvig-Waering, from Trinidad and Dominican Republic (Scorpiones, Buthidae).. *Acta Biologica Paranaense*.

[bibr21] Lourenço WR, Von Eickestedt VR (1983). Présence du genre *Microtityus* (Scorpiones, Buthidae) au Brésil.. Description de *Microtityus vanzolinii* sp. n.. *Revue Arachnologique*.

[bibr22] Morrone JJ (2001). Biogeografía de América Latina y el Caribe.. *M&T—Manuales & Tesis Sociedad Entomológica Aragonesa*.

[bibr23] Newlands G, Werger MJA (1978). Arachnida (except Acari).. *Monographiae Biologicae: Biogeography and ecology of Southern Africa*.

[bibr24] Prendini L, V Fet, Selden PA (2001). Substratum specialization and speciation in southern African scorpions: the Effect Hypothesis revisited.. Scorpions 2001. *In Memoriam Gary A. Polis*.

[bibr25] Prendini L, Huber BA, Sinclair BJ, Lampe KH (2005). Scorpion diversity and distribution in southern Africa: Pattern and process.. *African Biodiversity: Molecules, Organisms, Ecosystems*.

[bibr26] Polis GA (1990). *The biology of scorpions*..

[bibr27] San Martin PR (1968). Estudio preliminar sobre una nueva quetotaxia en escorpiones (Microtityus rickyi., Buthidae). Morfología y accion mecanica.. *Caribbean Journal of Science*.

[bibr28] Santiago-Blay J.A (1985). *Microtityus dominicanensis*: a new scorpion from Dominican Republic, West Indies ( Scorpiones, Buthidae).. *Entomological News*.

[bibr29] Santiago-Blay JA (1990). A new specimen of *Microtityus ambarensis*, fossil from Hispaniola: evidence of taxonomic status and possible biogeographic implications.. *Journal Arachnology*.

[bibr30] Santiago-Blay JA, Schawaller W, Poinar GO (1990). A new specimen of *Microtityus ambarensis* (Scorpiones, Buthidae), fossil from Hispaniola: evidence of taxonomic status and possible biogeographic implications.. *Journal Arachnology*.

[bibr31] Smith GT (1966). “Observations on the life history of the scorpion *Urodacus abruptus* Pocock (Scorpionidae), and an analysis of its home sites”.. *Australian Journal of Zoology*.

[bibr32] Stahnke HL (1966). Some aspects of scorpion behavior.. *Bulletin of the Southern California Academy of Sciences*.

[bibr33] Teruel R (2000). Una nueva especie de *Microtityus* Kjellesvig-Waering 1968 (Scorpiones, Buthidae) de Cuba oriental.. *Revista Ibérica. Aracnología*.

[bibr34] Teruel R (2001). Taxonomia y distribucion geografica de *Microtityus fundorai*
Armas 1974 (Scorpiones, Buthidae) en la provincia Santiago de Cuba, Cuba.. *Revista Ibérica Aracnología*.

[bibr35] Teruel R (2000). Una nueva especie de *Microtityus* Kjellesvig-Waering 1966 (Scorpiones: Buthidae) de Cuba oriental.. *Revista Ibérica de Aracnología*.

[bibr36] Teruel R (2001). Taxonomía y distribución geográfica de *Microtityus fundorai*
Armas 1974 (Scorpiones: Buthidae) en la provincia Santiago de Cuba, Cuba.. *Revista Ibérica de Aracnologia*.

[bibr37] Teruel R, Armas LF (2006). Un nuevo *Microtityus* Kjellesvig-Waering 1966 (Scorpiones: Buthidae) de Cuba oriental.. *Boletín de la Sociedad Entomológica Aragonesa*.

[bibr38] Teruel R, Infante LM (2007). Un nuevo escorpión del género *Microtityus* KjellesvigWaering 1966 (Scorpiones: Buthidae), de la región oriental de Cuba.. *Boletín de la Sociedad Entomológica Aragonesa*.

[bibr39] Vachon M (1977). Contribution â 1'étude des scorpions Buthidae du nouveau monde. I. complément âla connaissance de *Microtityus rickyi* Kj. W. 1956 de l'ile de la Trinité. II. Description d'une nouvelle espèce et d'un nouveau genre mexicains: Darchenia bernadettae. III. Clé de détermination des genres de Buthidae du nouveau monde.. *Acta Biológica Venezuélica*.

[bibr40] Warburg MR, Ben-Horin A (1978). “Temperature and humidity effects on scorpion distribution in Northern Isra'l”.. *Symposia of the Zoological Society of London*.

[bibr41] Yamaguti HY, Pinto-da-Rocha R (2006). Ecology of *Thestylus aurantiurus* of the parque Estadual da serra da cantareira, sao paulo, Brazil (Scorpiones, Bothriuridae).. *Journal of Arachnology*.

